# Structural and Material Optimization of a Sensor-Integrated Autonomous Aerial Vehicle Using KMU-3 CFRP

**DOI:** 10.3390/polym17162175

**Published:** 2025-08-08

**Authors:** Yerkebulan Nurgizat, Arman Uzbekbayev, Igor Fedorov, Andrey Bebenin, Andrey Karypov

**Affiliations:** 1 Research & Development Center “Kazakhstan Engineering” LLP, Astana 010000, Kazakhstanfigor.ole@gmail.com (I.F.); abebenin_77@mail.ru (A.B.);; 2 Institute of Telecommunications and Space Engineering, Almaty University of Power Engineering and Telecommunications Named Gumarbek Daukeev, Almaty 050062, Kazakhstan

**Keywords:** composite materials, aerospace engineering, carbon fibers, truss structures, finite element analysis, computational aerodynamics

## Abstract

This study addresses the selection and application of composite materials for aerospace systems operating in extreme environmental conditions, with a particular focus on high-altitude pseudo-satellites (HAPS). This research is centered on the development of a 400 kg autonomous aerial vehicle (AAV) capable of sustained operations at altitudes of up to 30 km. KMU-3’s microstructure, comprising high-modulus carbon fibers (5–7 µm diameter) in a 5-211B epoxy matrix, provides a high specific strength (1000–2500 MPa), low density (1.6–1.8 g/cm^3^), and thermal stability (−60 °C to +600 °C), ensuring structural integrity in stratospheric conditions. The mechanical, thermal, and aerodynamic properties of KMU-3-based truss structures were evaluated using finite element method (FEM) simulations, computational fluid dynamics (CFD) analysis, and experimental prototyping. The results indicate that ultra-thin KMU-3 with a wall thickness of 0.1 mm maintains structural integrity under dynamic loads while minimizing overall mass. A novel thermal bonding technique employing 5-211B epoxy resin was developed, resulting in joints with a shear strength of 40 MPa and fatigue life exceeding 10^6^ cycles at 50% load. The material properties remained stable across the operational temperature range of −60 °C to +80 °C. An optimized fiber orientation (0°/90° for longerons and ±45° for diagonals) enhanced the resistance to axial, shear, and torsional stresses, while the epoxy matrix ensures radiation resistance. Finite element method (FEM) and computational fluid dynamics (CFD) analyses, validated by prototyping, confirm the performance of ultra-thin (0.1 mm) truss structures, achieving a lightweight (45 kg) design. These findings provide a validated, lightweight framework for next-generation HAPS, supporting extended mission durations under harsh stratospheric conditions.

## 1. Introduction

The modern aerospace industry increasingly depends on composite materials to meet stringent demands for performance, weight reduction, and durability [1]. Traditional metal alloys, such as titanium, aluminum, and nickel, although offering high strength, are constrained by their high density and limited thermal resistance, accelerating the shift toward advanced composites. Carbon fiber-reinforced polymers (CFRPs) [2,3] have become fundamental due to their superior specific strength-to-weight ratios, constituting up to 50% of the structural mass of contemporary aircraft, including the Boeing 787 and Airbus A350. Ceramic matrix composites (CMCs) [4,5] are deployed in engines and thermal protection systems, withstanding temperatures exceeding 1500 °C, while metal matrix composites (MMCs) are utilized in components subjected to extreme thermal loads [6]. Despite significant advancements, critical challenges persist: CFRPs exhibit susceptibility to microcracking at cryogenic temperatures, CMCs remain inherently brittle, and the environmental sustainability of composite disposal continues to necessitate innovative solutions. These issues are particularly pronounced in the development of high-altitude pseudo-satellites (HAPS), where the stratosphere presents extreme conditions—including temperatures from −150 °C to +1500 °C [7], low atmospheric density, and elevated radiation levels—demanding materials that are simultaneously lightweight, resilient, and durable.

This study aims to develop and substantiate the selection of a composite material for an autonomous aerial vehicle (AAV) with a maximum take-off weight of 400 kg designed for HAPS applications, prioritizing minimal weight, a high mechanical strength, and resistance to stratospheric conditions.

The selection of a KMU-3 carbon fiber-reinforced polymer (CFRP) is driven by the synergy between its high-modulus carbon fibers and epoxy matrix, offering an elastic modulus of 300–600 GPa, tensile strength of 1000–2500 MPa, and thermal stability up to 600 °C [8,9]. Its low density (1.6–1.8 g/cm^3^) and radiation resistance make it ideal for stratospheric applications, where polymer composites must endure extreme conditions. This study leverages these properties to optimize lightweight aerospace structures for high-altitude missions.

Recent advances in high-altitude pseudo-satellites (HAPS) confirm the decisive importance of ultra-light composite airframes for achieving multi-week endurance with meaningful payloads. The Airbus Zephyr S (≈60 kg take-off mass, 25 m span, ≈5 kg payload) relies on a proprietary quasi-isotropic CFRP skin (HexPly^®^ M21; elastic modulus 185–225 GPa, density 1.55 g cm^−3^) [10]. The BAE Systems PHASA-35 (≈150 kg, 35 m span, ≈15 kg payload) uses Toray T1100G prepreg laminates (modulus ~370 GPa, density 1.78 g cm^−3^) in its spars and ribs [11]. The Google Loon super-pressure balloons reinforce the gondola frames with standard aerospace CFRP (modulus ~150 GPa) to withstand stratospheric gust loads [12].

While these platforms demonstrate the maturity of carbon composites for HAPS, their CFRP grades are tuned for extreme mass reduction rather than a high axial load-bearing capacity. The KMU-3 carbon fiber adopted in this study delivers an elastic modulus of 300–600 GPa and tensile strength up to 2.5 GPa at a comparable density (1.6–1.8 g cm^−3^), providing a 40–60% increase in the stiffness-to-weight ratio over the materials cited above. This performance margin allows the present work to employ 0.1 mm-thick KMU-3 tubes in a spatial truss, lowering structural mass yet preserving rigidity—an approach not documented in the peer-reviewed HAPS literature.

Alternative ceramic or ceramic hybrid composites (e.g., SiC/SiC, C/C-SiC) tolerate temperatures above 1500 °C, but their brittleness ($K_{IC}<5\;MPa\cdot m^{1/2}$), higher density, and complex processing render them unsuitable for thin-walled lightweight frames [13,14]. Consequently, KMU-3 offers the optimal combination of stiffness, strength, radiation resistance, and manufacturability required for a 400 kg autonomous aerial vehicle operating between −60 °C and +80 °C at altitudes around 30 km.

The specific objectives are as follows: to assess the feasibility of a KMU-3 carbon fiber-reinforced polymer (CFRP) for truss structures; to establish a joining methodology for ultra-thin-walled tubes (0.1 mm) [15,16]; to conduct computational fluid dynamics (CFD) and finite element method (FEM) analyses for aerodynamic and structural performance; and to validate simulation results through experimental prototyping. The novelty of this research lies in the application of KMU-3 within ultra-lightweight structures, the implementation of epoxy resin-based heat welding techniques, and the comprehensive optimization of design parameters. The methodological framework integrates material characterization, structural design, advanced numerical modeling, and empirical testing. The anticipated outcomes are expected to advance the development of high-performance AAVs and validate the proposed approaches for extreme aerospace environments.

## 2. Materials and Methods

### 2.1. Material Selection and Composite Characterization

When designing aerospace structures intended for extreme operating conditions, particular emphasis is placed on achieving a high mechanical strength, low weight, and enhanced structural rigidity while maintaining minimal mass.

The autonomous aerial vehicle (AAV) under development is required to sustain a maximum take-off mass of 400 kg, cruise at 70 m s^−1^, and remain airborne at 30 km altitude, while enduring gust load factors up to 1.8 g. Throughout its service life the structure must operate between −60 °C and +80 °C under cumulative UV exposure exceeding 600 MJ m^−2^ and deliver a fatigue life of at least 10^6^ cycles at 50% design load. To allocate sufficient margin for the batteries and payload, the load-bearing frame must not exceed 50 kg. These boundary conditions translate into the following material constraints: a specific modulus of no less than 150 GPa cm^3^ g^−1^ to limit aero-elastic deflection of the 40 m wing, a specific strength above 550 MPa cm^3^ g^−1^ for the primary longerons, an interlaminar shear strength of at least 35 MPa to prevent joint failure in 0.1 mm-thick trusses, temperature-dependent stiffness variation below ±10% over the operational range, and proven radiation resistance for multi-month stratospheric missions. A screening study of high-strength CFRP, aramid laminates, Al-Li alloys, MMCs, and SiC/SiC CMCs revealed that only high-modulus KMU-3 CFRP satisfies all of these criteria while remaining manufacturable as ultra-thin tubular members.

In the context of this study, the primary objective is to develop an unmanned aerial vehicle (UAV), specifically an autonomous aerial apparatus (AAA), capable of sustained operation at altitudes up to 30 km, with a total mass not exceeding 400 kg, through the optimal selection of composite materials. Meeting these stringent mass and strength requirements necessitates the use of materials characterized by a high specific strength, thermal stability, and corrosion resistance [17,18,19].

The selection of manufacturing processes is influenced by several critical factors, including component geometry, material availability, cost efficiency, quality standards, production timelines, available technical expertise, and anticipated production volume. Consequently, a significant proportion of the project’s cost and resources are allocated to the development and optimization of composite materials [20,21], which continue to be at the forefront of current research and technological innovation.

**Figure 1:**
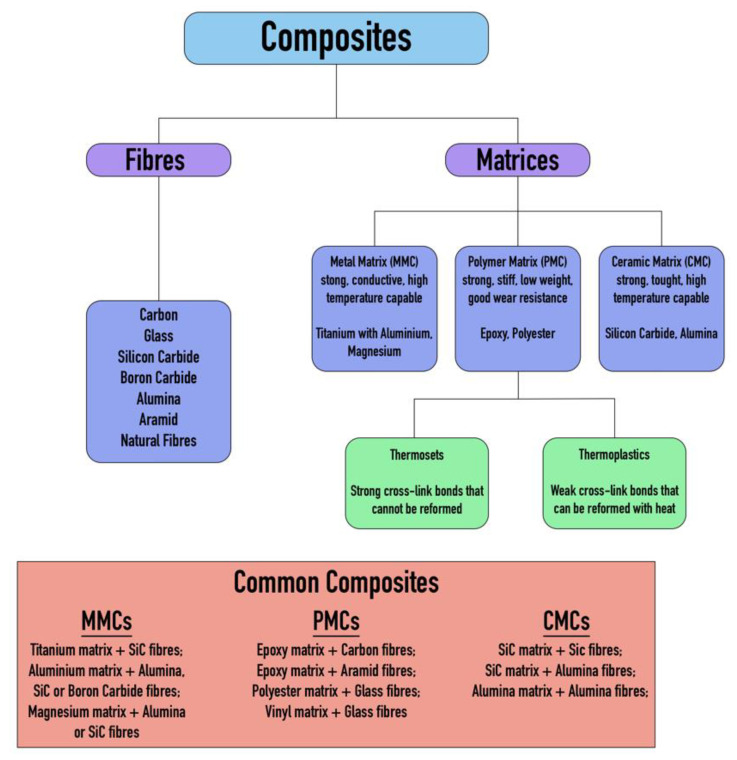
Definition of composite materials [23].

Composite materials are conventionally fabricated using fibrous reinforcements—such as carbon or glass fibers—that provide the necessary tensile strength and stiffness, embedded within a matrix material that imparts shape, structural support, and impact resistance [22]. The overall performance characteristics of the composite depend significantly on the properties and configuration of both the reinforcement fibers and the matrix material. An overview of the interaction between these two components and their influence on the final material properties is illustrated in Figure 1.

Among polymer-based composites, CFRPs [24] are distinguished by their high specific strength and stiffness, making them essential for building lightweight yet durable structural elements. FRPs, particularly those reinforced with glass fibers, offer excellent corrosion resistance and are more cost-effective, although they fall short of CFRPs in mechanical performance. Aramid composites, such as Kevlar, are frequently used for mechanical impact protection due to their superior toughness. MMCs, like aluminum matrices reinforced with silicon carbide, combine thermal conductivity with mechanical strength, which is advantageous for components exposed to significant heat loads. CMCs are employed in applications requiring resistance to extremely high temperatures, such as thermal protection systems, though their brittleness necessitates additional engineering measures. Hybrid composites, which integrate different types of fibers or matrices, allow for an optimized balance of properties tailored to specific operating conditions [25,26].

A comparative analysis and classification of traditional metal alloys, polymer composites, ceramics, as well as modern CFRP, FRP, and aramid composites is presented in Table 1.

The selection of high-modulus CFRP for a 400 kg autonomous aerial vehicle designed for altitudes up to 30 km is justified by its high stiffness (modulus of elasticity of 300–600 GPa), low density (1.6–1.8 g/cm^3^), sufficient tensile strength (1000–2500 MPa), and resistance to stratospheric conditions (temperature, UV radiation, corrosion). These properties enable the creation of a lightweight, rigid, and durable structure capable of withstanding aerodynamic loads and supporting long-duration autonomous flight. Other materials, such as aramid composites (e.g., Kevlar) or FRP (e.g., fiberglass), may be used in combination with CFRP to enhance impact resistance, but CFRP remains the optimal choice for load-bearing structures.

The performance of KMU-3 CFRP is driven by its microstructure, featuring high-modulus carbon fibers (5–7 µm diameter) in a 5-211B epoxy matrix with a 60% fiber volume fraction. This structure optimizes load transfer, ensures low water absorption (0.02%), and enhances interfacial bonding, validated by ASTM D3039 standards [20], making KMU-3 suitable for stratospheric conditions.

The mechanical properties of various CFRP grades are summarized in Table 2.

All numerical properties in Table 2 correspond to the 0° fiber direction. The corrosion and water uptake indices follow the 1–10 ranking [30] (10 = best). The water uptake values consider 24 h immersion at 23 °C; the cost figures are the 2024 aerospace prepreg averages.

A comparative analysis based on the above table indicates that high-modulus CFRPs (KMU), with an elastic modulus of 300–600 GPa and tensile strength of 1000–2500 MPa, are ideal for load-bearing structures such as wings and fuselage due to their exceptional stiffness. According to studies [38,39,40,41,42] supporting the performance characteristics of KMU-3, this particular grade demonstrates an optimal balance of properties and is well suited for stratospheric conditions. Manufacturing considerations also played a crucial role in the selection of KMU-3. Although its cost is classified as high—attributable to the use of high-quality carbon fibers and advanced fabrication methods such as autoclave molding—this is offset by lower operational costs. The lightweight nature of KMU-3 significantly reduces energy consumption, which is critical for an autonomous vehicle reliant on solar power. In comparison, heat-resistant CFRPs (very high cost) and standard polymer composites (very low cost) are either prohibitively expensive or fail to meet the necessary stiffness and thermal resistance requirements for operations at 30 km altitude.

In selecting KMU-3 for the 400 kg autonomous aerial vehicle designed for stratospheric deployment at 30 km altitude, key factors such as long-term durability, radiation resistance, and production-related aspects (cost and availability) were considered. As a high-modulus CFRP, KMU-3 offers an elastic modulus of 300–600 GPa, which translates to excellent fatigue resistance, allowing the material to endure cyclic loading over extended periods—a critical requirement for HAPS platforms that may remain airborne for weeks or even months. Moreover, KMU-3 exhibits a high specific strength (1000–2000 MPa) at a low density of 1.6–1.8 g/cm^3^, minimizing the structural mass and reducing energy demands.

At 30 km altitude, the vehicle is exposed to intense ultraviolet (UV) radiation and cosmic rays, including protons and gamma radiation, due to the diminished protection of the ozone layer. KMU-3 demonstrates strong radiation resistance, particularly when used in combination with protective coatings such as epoxy resins with UV stabilizers, as confirmed by research [41]. This ensures the material retains its mechanical properties under extreme stratospheric conditions, where ambient temperatures fluctuate from −60 °C to +80 °C due to solar heating. By contrast, Kevlar, despite its high impact toughness (125 kJ/m^2^), is vulnerable to UV degradation unless adequately shielded, while MMCs are susceptible to radiation-induced corrosion.

### 2.2. Truss Structure Design and Element Joining Technology

This section discusses the principles of designing composite truss structures for aerospace applications, as well as the joining technologies that ensure the strength and durability of the overall structure.

The structural concept is governed by a clear set of quantitative requirements. The autonomous aerial apparatus must not exceed a maximum take-off mass of 400 kg, of which 100–160 kg is reserved for the payload and batteries, leaving no more than 50 kg for the load-bearing frame. During cruising at 30 km altitude (air density ≈ 0.018 kg m^−3^) and 70 m s^−1^ true airspeed, the airframe has to withstand gust load factors up to ±1.8 g while limiting wing-tip deflection to 0.4 m (1% of the 40 m span). All primary members therefore require a specific axial stiffness of at least 150 GPa cm^3^ g^−1^. The environmental limits include a temperature window of −60 °C to +80 °C and a cumulative ultraviolet fluence of ~600 MJ m^−2^ over a three-month mission. The bonded joints must deliver a minimum inter-laminar shear strength of 35 MPa and survive 10^6^ load cycles at 50% design stress, providing a global ultimate-strength safety factor of 2.0 in line with CS-25 guidance for high-altitude UAVs. These constraints dictate the choice of KMU-3 CFRP, the 0.1 mm wall thickness for longerons and diagonals, and the thermal-bonded joining technology described below.

In the design of a truss structure and the development of the joining technology for a 400 kg AAA intended to operate at an altitude of 30 km using high-modulus CFRP (KMU-3), it is essential to consider the extreme conditions of the stratosphere, such as low temperatures (−60 °C), solar heating (up to +80 °C), radiation, and low air density. The truss structure must be lightweight, rigid, and strong, while the joining method must be reliable and contribute minimally to the overall mass.

#### 2.2.1. Truss Structure Design for AAA

Material selection was one of the key requirements in designing the truss structure: KMU-3, with an elastic modulus of 300–600 GPa, a tensile strength of 1000–2500 MPa, a density of 1.6–1.8 g/cm^3^, and thermal resistance up to 600 °C. Additionally, a 0.1 mm shell was incorporated to ensure structural integrity under dynamic loads. The design and simulation of the truss structure were carried out using ANSYS Mechanical R1 (22.1). Table 3 provides the detailed specifications of the truss geometry, including the materials, dimensions, configuration, and total mass.

**Figure 2:**
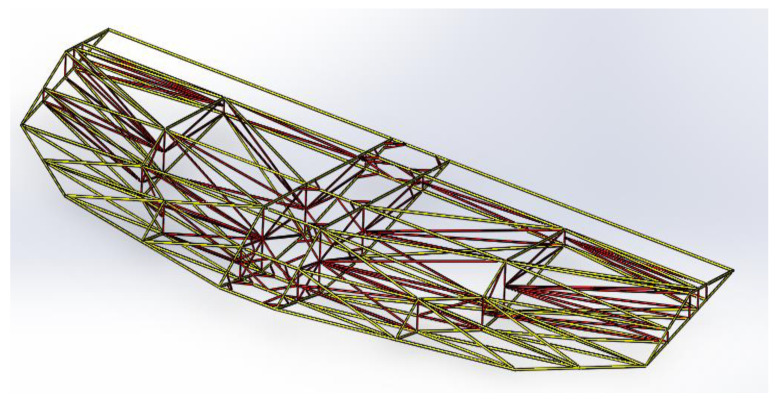
Truss structure: top view.

**Figure 3:**
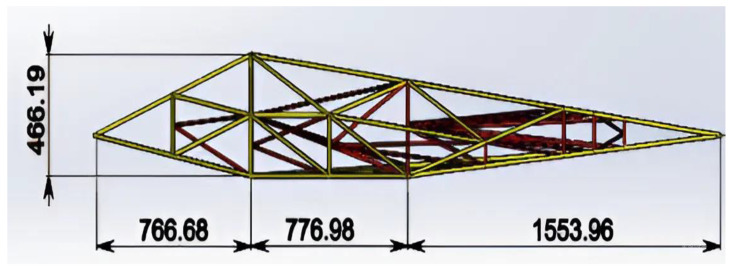
Truss structure: side view.

Based on the specifications presented in Table 3, Figure 2 shows the top view of the truss structure, while Figure 3 presents a side view and a simplified diagram of the rod connections. This truss configuration enables the optimal distribution of external loads across the structural elements and significantly reduces weight compared with conventional metallic systems.

**Figure 4:**
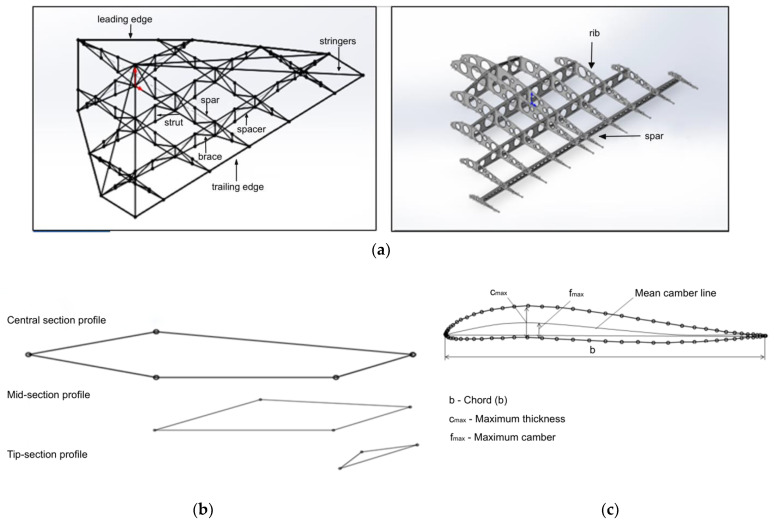
Structural design solutions for aircraft configurations with key parameters. (**a**) Truss structure of the aircraft; (**b**) profiles of the truss structure with geometric and aerodynamic twist; (**c**) S-shaped profile for a tailless aircraft.

A truss structure with triangular cells is considered optimal for UAVs [43], as it provides maximum stiffness with minimal weight. As shown in Figure 4, the structure will consist of thin-walled KMU-3 tubes (diameter 10–20 mm, wall thickness 0.1 mm) forming a spatial truss for the wings and fuselage. Further details can be seen in Figure 4, which illustrates the aircraft wing structure, including its load-bearing frame (Figure 4a), the profile shape variation along the wingspan (Figure 4b), and the aerodynamic profile geometry (Figure 4c). This configuration minimizes deformation, which is critical for large wingspans (30–40 m) required for flight in rarefied atmospheric conditions.

To estimate the mass of the truss structure for an AAA with a total mass of 400 kg and designed to operate at an altitude of 30 km, the following input parameters are used: material density ρ=1.7 g/cm³, longeron diameter $d_{l}=1.5\;cm$, diagonal diameter $d_{r}=1.0\;cm$, wall thickness t=0.01 cm, lengths $L_{l}=300,000\;cm$, $L_{r}=250,000\;cm$, number of joints N=1200, and joint mass $m_{joint}=0.5\;g$.

The mass is calculated using the following equations:

(1) m = ρ × V,

(2) V=A×L,

(3) A=π×d×t.

Mass of longerons:

$$A_{l}=\;\pi \;\times \;1.5\;\times \;0.01\;\approx \;0.0471\;[cm^{2}];$$

$$V_{l}=0.0471\times 300,000\;\approx \;14,130\;[cm^{3}];$$

$$m_{l}=1.7\times 14,130\;\approx \;24,021\;g=24.021\;[kg].$$

Mass of diagonals:

$$A_{r}=\;\pi \;\times \;1.0\;\times \;0.01\;\approx \;0.0314\;[cm^{2}];$$

$$V_{r}=0.0314\times 250,000\;\approx \;7850\;[cm^{3}];$$

$$m_{r}=1.7\times 7850\;\approx \;13,345\;g=13.345\;[kg].$$

Total tube mass:

$$m_{tubes}=24.021+13.345=37.366\;[kg];$$

Mass of joints:

$$m_{joints}=1200\times 0.5=600\;g=0.6\;[kg];$$

Total initial mass:

$$m_{truss}=37.366+0.6=37.966\;[kg];$$

Final truss mass (with 10% margin):

$$m_{final}=37.966\times 1.1=41.763\;kg\approx 42\;\left[kg\right].$$

The KMU-3 CFRP truss structure ensures a high stiffness due to its elastic modulus of 300–600 GPa and triangular geometry, while maintaining a low structural mass (under 50 kg). This supports effective flight in low-density stratospheric conditions. Additionally, KMU-3 exhibits excellent resistance to temperature fluctuations and radiation, thanks to its high thermal tolerance (up to 600 °C) and improved radiation resistance when protected with surface coatings.

#### 2.2.2. Element Joining Technology

**Figure 5:**
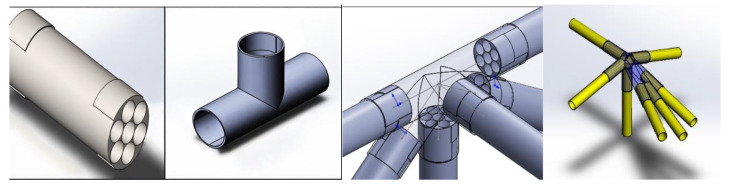
Coupling, structural tube section, and thermal coupling joint of elements.

One of the key challenges in designing a CFRP truss structure is the reliable joining of composite tubes. Studies [44] indicate that the most effective method for composite rods is fitting-based thermal bonding. As shown in Figure 5, several critical factors must be considered when applying this technique: the tubes are manufactured with a precision of ±0.05 mm, ensuring a gap of 0.1–0.2 mm, and their surfaces are treated with sandblasting and coated with primer to enhance adhesion. A low-viscosity epoxy resin (viscosity: 200 mPa·s) containing 5% carbon microfibers by mass is then injected through micro-channels under 0.5 bar pressure, in accordance with large-scale resin infusion technology (Section 2.2.1). Curing is carried out outside the autoclave at 120 °C for 2 h, minimizing the production costs and avoiding the thermal degradation of KMU-3 (which tolerates up to 600 °C). Ultrasonic inspection is used for quality control to detect bonding defects and confirm a shear strength of 40 MPa and fatigue life of 10^6^ cycles under 50% load of the maximum tensile strength.

The 5-211B epoxy resin, a low-viscosity thermoset (200 mPa·s) with 5% carbon microfibers, enhances adhesion to KMU-3’s carbon fibers through a high cross-linking density, as supported by [45]. Its rheological properties ensure uniform infusion during bonding, minimizing voids and optimizing interfacial bonding for stratospheric durability.

To protect the joints from stratospheric conditions, an epoxy coating with UV stabilizers is applied at a thickness of 0.05 mm, as noted in Section 2.2.1. This prevents material degradation under radiation exposure. The mass of a single joint is only 0.5 g, resulting in a total joint mass of just 0.6 kg, which aligns with previous calculations and ensures minimal structural weight. The manufacturing process of the truss structure is optimized through automated resin application and infusion, reducing the production costs and justifying the high material cost of KMU-3 (see Table 2). Moreover, out-of-autoclave curing simplifies the process further.

Adherence to these specifications prevents delamination and other common failure modes in composites while ensuring high fatigue resistance.

### 2.3. Numerical Methodology for Aerodynamic and Structural Analysis

In this study, the use of CFD for aerodynamic analysis and FEM for structural analysis is essential for the integrated design of an AAA intended for high-altitude unmanned aerial operations in stratospheric environments. These methods enable the comprehensive analysis and optimization of both aerodynamic and structural characteristics, which are critical to achieving a light weight, strength, and efficiency in the overall design.

Both approaches are integrated into a unified workflow: CFD is used to determine the aerodynamic loads, which are subsequently applied in FEM simulations to evaluate structural integrity. This combined strategy ensures a balance between aerodynamic performance and structural reliability, which is particularly important for AAA, where mass (≤400 kg) and long-term durability are of paramount importance. CFD assists in selecting the optimal wing configuration, while FEM validates that the chosen materials and joints can withstand the expected loads—ensuring both lightness and robustness.

The application of CFD and FEM also eliminates the need for costly physical testing, which is especially valuable in AAA development where prototyping at 30 km altitude is impractical. These simulation-based methods support iterative design improvements and confirm that the KMU-3 CFRP structure—with a 0.1 mm wall thickness and truss segments up to 5 m in length—achieves the target mass and strength requirements while sustaining a cruise speed of 70 m/s with 13 kW of solar power input.

#### 2.3.1. Aerodynamic Analysis Methodology (CFD)

CFD serves as a critical tool in the aerodynamic design of the AAA for high-altitude unmanned platforms operating at 30 km in the stratosphere. Under extreme atmospheric conditions—including a low air density (0.0184 kg/m^3^), ambient temperature of −46.5 °C, and exposure to radiation—CFD enables the accurate modeling of aerodynamic characteristics such as lift, drag, and stability to support optimal wing design.

In this study, CFD is employed to evaluate various wing configurations (e.g., delta, rhomboid, flying wing) to ensure efficient flight at a cruise speed of 70 m/s with minimal energy consumption. The core governing equations are the Navier–Stokes equations, which are solved numerically using finite difference or finite volume methods. CFD simulation identifies critical regions of aerodynamic stress, refines structural shapes, reduces drag, and enhances stability—all without the need for costly full-scale physical testing.

The aerodynamic simulations are conducted under the following standard atmospheric conditions at an altitude of 30 km:

- $\rho =0.0184\;kg/m^{3}$—air density;
- T=226.65 K(−46.5 °C)—temperature;
- $\mu =1.48\;\times 10^{-5}\;kg/(ms)$—dynamic viscosity;
- V=70 m/s—flight speed;
- M=0.232—Mach number, where a=301.87 m/s is the speed of sound; although subsonic, the proximity to Mach 0.3 necessitates compressibility considerations;
- Re≈435,000—Reynolds number (based on wing chord length L = 5 m), computed as $Re=\frac{\rho VL}{\mu }$; indicates transitional flow, making turbulence modeling critical;
- $S=200\;m^{2}$—wing area.

To determine the optimal aerodynamic configuration for the AAA, several wing designs—including wedge-shaped, delta-wing, and flying wing with internal truss structure—were modeled. For each design, 3D CAD models were developed, and the flow behavior was simulated using the ANSYS Fluent R1 (22.1) software package.

**Figure 6:**
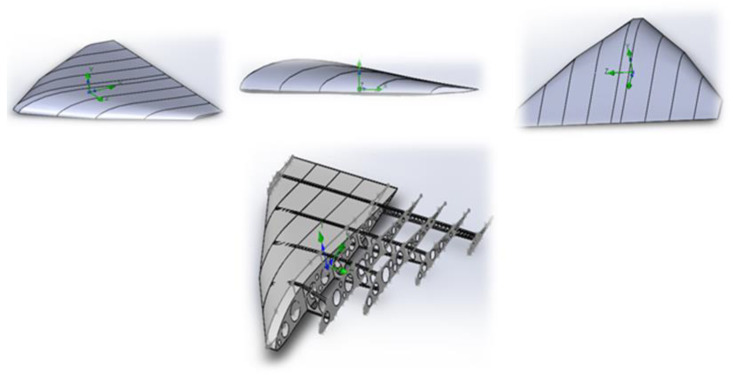
Solid 3D model of the device.

Figure 6 shows an example of a three-dimensional model of the AAA, illustrating its overall geometry and the possible placement of the truss elements.

A structured hexahedral mesh is employed to ensure high accuracy in the boundary layer region. Inflation layers with $y^{+}\approx 1$ are used to resolve the near-wall flow accurately. The mesh is refined in regions with high gradient concentrations, such as the leading and trailing edges. A mesh convergence study is performed to verify that the simulation results are independent of the grid resolution.

In general, the solution of the flow problem around an aircraft involves the numerical solution of the Navier–Stokes Equation (4) under specified initial and boundary conditions [35].

(4) $$\frac{\partial \vec{U}}{\partial t}+\left(\vec{U}*\nabla \right)\vec{U}=F-\frac{1}{\rho }grad\;p+v\cdot ∆\vec{U},\;\frac{\partial p}{\partial t}+divp\vec{U}=0$$

In the absence of body forces and assuming ρ = const, Equation (1) can be written as follows:

$$\frac{\partial \vec{U}}{\partial t}+\left(\vec{U}\cdot \bar{V}\right)\cdot \vec{U}=-\frac{1}{\rho }gradp+v\cdot ∆\vec{U},\;divU=0$$

The boundary and initial conditions include no-slip or slip conditions for the velocity vector at solid boundaries, pressure values specified at domain boundaries, and flow velocity conditions normal or at an angle to the boundary. In addition, a combination of these conditions can be applied, such as specified pressure and velocity values at the boundary.

The lift force (Y) depends on the circulation of velocity (Γ) and is given by Joukowski’s theorem for a wing section of length L (along the span) in a plane-parallel flow of an ideal incompressible fluid, shown as follows in Equation (5):

(5) Y=ruΓL,

where r—the density of the fluid and u—the free stream velocity.

The circulation (Γ) has the dimension [u × l], and it can be shown that the lift force can be expressed as follows:

(6) $$Y=C_{y}\frac{\rho V^{2}}{2}S,$$

where S is the characteristic reference area of the body (wing planform area, given by L × b, where b is the chord length of the airfoil) and $C_{Y}$ is the dimensionless lift coefficient, which generally depends on aerothermodynamic conditions, including the shape of the body, its orientation in the flow, and the Reynolds (Re) and Mach (M) numbers.

In addition to the lift force, the airfoil also experiences a drag force, which can be expressed as follows:

(7) $$X\;=\;C_{X}S_{\rho }V/2.$$

The drag coefficient $C_{X}$ and the drag force X primarily consist of the following four main components:

- $C_{X}$, wave ${(C}_{X}$, w)—Wave drag, which appears at Mach numbers close to the critical Mach number (M ≈ 0.8).
- $C_{X}$, friction ($C_{X}$, tr)—Friction drag, caused by air resistance against the aircraft surface.
- $C_{X}$, pressure ($C_{X}$, vorticity drag)—Also referred to as vortex drag, resulting from pressure differences around the body.
- $C_{X}$, induced ($C_{X}$, i)—Induced drag, occurring due to flow deflection, including wing-tip vortices and pressure differences between the upper and lower surfaces of the wing.

From Equations (6) and (7), the following dependency can be derived in Equation (8):

(8) $$\frac{Y}{X}=\frac{C_{y}}{C_{x}}=\varepsilon ,$$

where *ε*—the aerodynamic efficiency.

**Figure 7:**
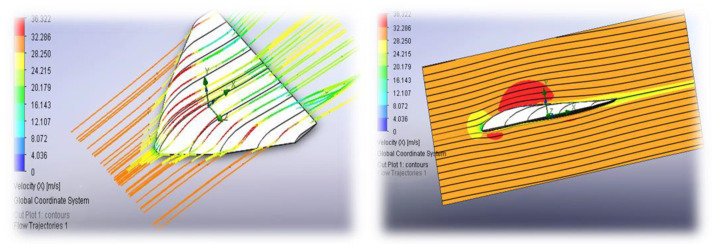
Numerical simulation of flow velocities around the vehicle: flow model and turbulence model.

The results of the numerical simulation of flow velocities around the apparatus are shown in Figure 7. Numerical simulations were performed using the following boundary conditions: a free-stream flow was applied at the far-field boundary with a velocity of 70 m/s, temperature of 226.65 K, and static pressure of 1197 Pa. A no-slip and adiabatic wall condition was imposed on the wing surface. To optimize computational efficiency, geometric symmetry was utilized by modeling only half of the configuration.

Second-order upwind schemes were used for the convective terms, while central dif-ferencing was applied for the diffusive terms. A density-based solver suitable for compressible flow was employed. Solution convergence was monitored through residuals falling below 10^–5^ and the stabilization of lift, drag, and moment coefficients.

Steady-state simulations were conducted for angles of attack ranging from 0° to 15° in 2° increments to generate lift and drag polars. For each wing configuration, lift, drag, and moment coefficients were computed. Pressure and velocity distributions were analyzed, along with flow visualization via streamlines and vortex structures. In particular, for delta-wing configurations, vortex-induced lift becomes significant at high angles of attack, highlighting the importance of an integrated approach to material selection and the need for further research on hybrid composite materials.

**Figure 8:**
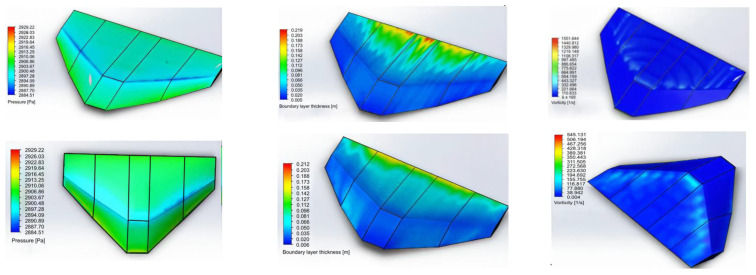

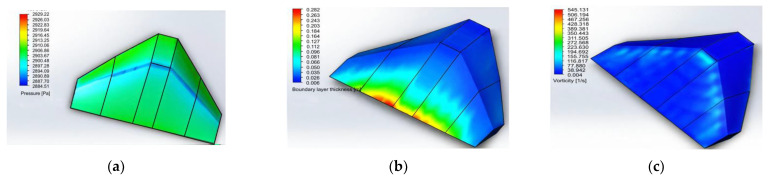
Study of static, dynamic, and overload loads on the aircraft. (**a**)—pressure distribution; (**b**)—boundary layer thickness; (**c**)—turbulence.

As a visual representation of the numerical simulation results, Figure 8 shows an example of the study of static and dynamic loads and overloads applied to the aircraft. Figure 8 shows the following:

- The pressure distribution on the surface of the airframe and the main truss elements (Figure 8a).
- The variation in local stresses in the structure under static loading (Figure 8b).
- The potential overloads occurring during maneuvers or wind gusts (Figure 8c).

The CFD analysis aims to ensure that the generated lift force matches the vehicle’s weight (3920 N for 400 kg). The calculations show that achieving this balance at a cruise velocity$\;V=70\;\frac{m}{s}$ and wing area$\;S=200\;m^{2}$ requires a lift coefficient of approximately $C_{L}\approx 0.435$.

This is derived from the following lift Equation (9):

(9) $$Y=0.5\times \rho \times V^{2}\times S\times C_{L}$$

Substituting known values as follows:

$$3920=0.5\times 0.0184\times 70^{2}\times 200\times C_{L},$$

$$C_{L}=\frac{3920}{\left(0.5\times 0.0184\times 4900\times 200\right)}\approx 0.435.$$

However, using a slightly higher $C_{L}=0.5$ would yield a lift force of approximately 4508 N, indicating a margin of excess lift or the possibility of adjusting the angle of attack for optimized cruise performance. This highlights the importance of an integrated design approach to enhance the aerodynamic efficiency of aerospace systems through careful material and geometry selection.

The expected results for the delta-wing configuration are as follows:

- $\alpha =0{}^{\circ}:C_{L}\approx 0;C_{D}\approx 0.01$ (parasitic drag);
- $\alpha =5{}^{\circ}:C_{L}\approx 0.4;C_{D}\approx 0.015$;
- $\alpha =10{}^{\circ}:C_{L}\approx 0.8;C_{D}\approx 0.03$ (due to vortex-induced lift).

The rhomboid wing design may yield a lower $C_{Lmax}$ but offers reduced drag at lower angles of attack. An analysis of the lift-to-drag ratio (L/D) is used to identify the most aerodynamically efficient configuration.

A three-level grid independence study was performed (Table 4). The coarse mesh contained 1.23 × 10^6^ hexahedral cells with a first layer height of 0.030 mm; the medium mesh 3.82 × 10^6^ cells at 0.015 mm; and the fine mesh 5.57 × 10^6^ cells at 0.010 mm. Twenty inflation layers with a growth rate of 1.15 produced y^+^ ≤ 1 over 90% of the wing surface and y^+^ < 3 elsewhere. The lift and drag coefficients differ by less than 2.5% between the medium and fine grids; consequently the 3.82 M-cell mesh was adopted for production runs. The steady-state pressure field exported from ANSYS Fluent was mapped into ANSYS Mechanical via the External Data interface. Perturbing this pressure field by ±3% changed the peak von Mises stress by only 1.6%, well inside the ultimate safety factor of 2.0.

The computational results suggest that unconventional configurations, when operated at optimal angles of attack, offer high aerodynamic efficiency. Additionally, the structural mass is reduced through the use of lightweight CFRP tubing, which is particularly advantageous for high-altitude operations. Similar findings were reported by NASA laboratories, emphasizing the benefits of lightweight composite load-bearing systems.

The CFD results are validated through comparisons with analytical models (e.g., Zhukovsky’s lift theorem) and data from analogous HAPS designs. Due to the lack of empirical data under stratospheric conditions, this study relies on well-established correlations and findings from prior research, such as analyses of high-altitude airships.

The simulation results confirm that CFRP is the only material capable of meeting the stringent strength-to-weight requirements without additional corrosion protection, even under harsh environmental conditions. CFD thus plays a critical role in the design of AAAs, enabling aerodynamic optimization [46] for sustained flight at 30 km altitude. The simulations verify that a wing with $C_{L}$ ≈ 0.435–0.5 produces sufficient lift (≈3920 N) to balance vehicle weight while minimizing drag. When coupled with FEM analysis, the KMU-3 truss structure is confirmed to withstand mechanical loads with adequate safety margins while keeping the total mass below 400 kg.

This approach reduces the reliance on costly physical testing and establishes a solid foundation for the development of reliable and efficient HAPS platforms for extended-duration stratospheric missions.

According to the computational experiment, a non-traditional configuration with an optimal angle of attack provides high aerodynamic performance indicators. Additionally, the structural mass is reduced due to the use of carbon fiber tubes, which is particularly significant for high-altitude flights. Similar results were obtained in NASA laboratories, where the advantage of lightweight composite load-bearing systems was emphasized.

As demonstrated above, the only material capable of meeting the stringent requirements for mass and strength under such conditions is CFRP. Based on such studies, specific requirements for the properties of composites (elastic modulus, compressive strength limits, impact toughness, etc.) are formulated, which were taken into account in the design of the AAA.

#### 2.3.2. Calculation of Strength Characteristics (FEM)

This section examines the finite element method (FEM) used to analyze the structural strength characteristics of composite materials. A finite element model has been developed in ANSYS Mechanical to determine the stress–strain state of tubular elements and their connection zones (fitting-based thermal bonding). This method allows the simulation of the stress and deformation distribution in composite materials, taking into account the complex loading conditions of the KMU-3 carbon fiber composite, as well as the adhesive in the joints.

The objective is to determine the minimum required wing area (approximately 200 m^2^) and mass (no more than 400 kg) to meet operational requirements, including powering engines and on-board systems using solar energy, maintaining a reasonable battery reserve (up to 40% of total mass), achieving a cruise speed of 70 m/s, and ensuring a propulsion power of 13 kW with 95% efficiency.

The wing is a thin-walled structure made of KMU-3 carbon fiber composite with a wall thickness of 0.1 mm. For simplicity, the wing shape is assumed to be rectangular with a span of 40 m and a chord of 5 m, giving an area of 200 m^2^. The internal structure is reinforced with a truss system of carbon fiber tubes. The total mass of the wing does not exceed 400 kg, of which up to 40% (160 kg) is accounted for by the solar panels and batteries, evenly distributed over the surface.

The material properties of KMU-3 are as follows:

- Density: 1450 kg/m^3^;
- Tensile strength along fibers: 1100 MPa;
- Young’s modulus along fibers: 180 GPa;
- Young’s modulus across fibers: 9 GPa;
- Shear modulus: 5.1 GPa.

Considering the operational requirements, the wing must provide sufficient power for the propulsion system, which operates at 13 kW with 95% efficiency, giving an effective power output of approximately 13.7 kW after losses. The solar panels, which cover a significant part of the 200 m^2^ wing area, generate energy during the day, while batteries (weighing up to 160 kg) maintain operation at night. The aircraft achieves a cruise speed of 70 m/s at an altitude of about 30 km, where the air density is extremely low (about 0.018 kg/m^3^).

The aerodynamic lift force is calculated to ensure stable flight at the specified speed. The lift formula is given by Equation (10) as follows:

(10) $$L=\frac{1}{2}\rho V^{2}SC_{L},$$

where

- ρ = 0.018 kg/m^3^—air density at an altitude of 30 km;
- V = 70 m/s—cruise speed;
- S = 200 m^2^—wing area;
- $C_{L}$ = 0.5—lift coefficient (a typical value for a wing).

The values are substituted as follows:

$$L=\frac{1}{2}\times 0.018\times 70^{2}\times 200\times 0<5\approx 4410\;\left[N\right].$$

This force is evenly distributed across the wing and balances the weight of the structure (400 kg × 9.81 m/s^2^ ≈ 3924 N), confirming that a 200 m^2^ wing area is sufficient.

The weight load from the 400 kg mass, including 160 kg of batteries and solar panels, is also distributed across the wing. The total load consists of a combination of aerodynamic lift force and weight force.

The FEM simulations employ an orthotropic material model, with fiber orientation aligned at 0°/90° in the longerons to maximize axial strength, and ±45° in the diagonal members to ensure shear and torsional resistance. The matrix material used is epoxy resin 5-211B, which provides strong adhesion and enhanced radiation resistance.

Thermal effects are taken into account, with the elastic modulus increasing by approximately 5% at −60 °C and decreasing by about 10% at +80 °C. These variations highlight the importance of a comprehensive material selection strategy and suggest the potential of hybrid composites for improving the performance and reliability of aerospace systems under extreme conditions.

For structural analysis, a 3D wing model with a truss structure is created. The thin-walled sections are modeled as shell elements, while the truss system is represented by beam elements. The anisotropic properties of the KMU-3 carbon fiber composite are assigned according to its mechanical characteristics. Boundary conditions include fixing the wing at its attachment points to the fuselage. Loads are applied as distributed aerodynamic forces and weight loads.

**Figure 9:**
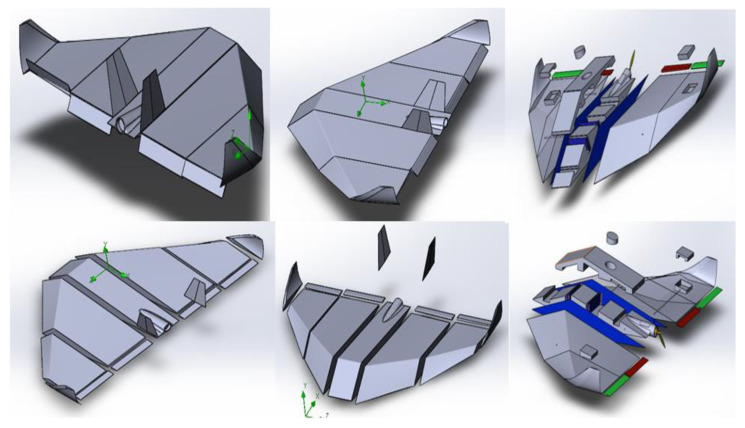
Three-dimensional models of AAA elements.

Figure 9 shows the 3D models of the selected AAA elements during assembly. The calculation results confirmed that with the correct choice of wall thickness (approximately 0.1 mm) and material (KMU-3), the required strength margin is achieved while maintaining the mass and dimensional constraints within 400 kg.

**Figure 10:**
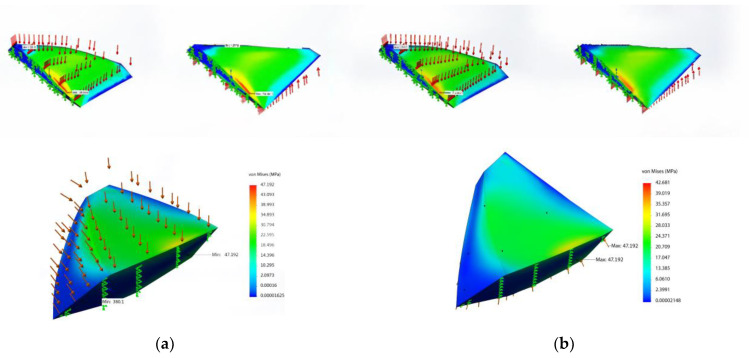
Simulated wing: (**a**) unconventional and (**b**) traditional Configurations.

**Figure 11:**
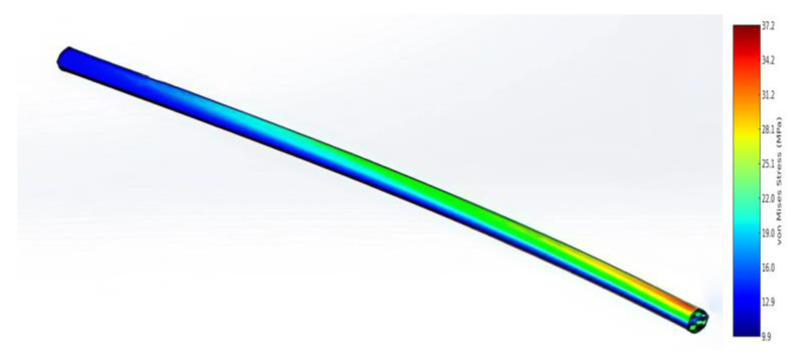
Stress distribution diagram in a circular section tube with one end fixed.

During the FEM analysis (Figure 10), the stress and deformation distribution was determined for the blade in both the traditional and unconventional configurations (delta and rhombic profiles). In addition, the behavior of a tubular element with one end fixed was analyzed (Figure 11). The red and yellow zones indicate areas of maximum stress, while the blue zones correspond to areas of minimum stress. The maximum stress was found to be 37.23 MPa and the minimum stress was 9.9 MPa.

Following the FEM analysis in ANSYS Mechanical, the following characteristics were obtained:

- The maximum stress in the structure is about 50 MPa, which is significantly lower than the tensile strength of the carbon fiber composite (1100 MPa).
- The maximum wing deflection is about 0.5 m, which is acceptable for a 40 m wingspan.
- The total structural mass is less than 400 kg, including a battery reserve of up to 160 kg.

The finite element analysis confirms that a 200 m^2^ wing with a total mass of no more than 400 kg made of KMU-3 carbon fiber composite meets the operational requirements. It can withstand aerodynamic loads at a cruise speed of 70 m/s, power the propulsion system (13 kW) from solar energy [41], and maintain a reasonable battery reserve (up to 40% of total mass). The structure has sufficient strength and stability for flights at altitudes of up to 30 km.

### 2.4. Optimization of Truss Structure Using the Gradient Method

To achieve an optimal balance between mass, strength, and stiffness in the KMU-3-based truss structure, an optimization process was carried out using the gradient method in ANSYS Workbench R1 (22.1) and AutoCAD 2022. This approach enabled the systematic improvement of structural parameters by minimizing the weight while meeting stringent operational requirements.

The objective of the optimization is to minimize the structural mass (M) while maintaining the ability to withstand aerodynamic and weight loads. The problem is formulated as follows: the objective function aims to minimize mass (M), with design variables including tube diameter (ranging from 10 to 15 mm for longerons and 8–12 mm for diagonals), fiber orientation angles (0°/90° for longerons and ±45° for diagonals, with a variation margin of ±10°), and joint placement defined by node spacing in the range of 1–2 m. Constraints include a maximum stress $\sigma_{max}$ below 1100 MPa (the tensile strength limit of KMU-3), maximum deflection (δ) not exceeding 0.4 m (1% of the wingspan), shear stress at joints below 40 MPa, and total mass not exceeding 400 kg, including a 160 kg payload.

The gradient method was chosen due to its efficiency in solving problems with continuous variables and smooth dependencies, as is typical in FEM analysis. The method was implemented in AutoCAD, integrated with FEM simulations. The algorithm uses the derivatives of the objective function (mass) with respect to the design variables (tube diameter, fiber angles, and node spacing) to determine the direction of steepest descent in mass.

**Figure 12:**
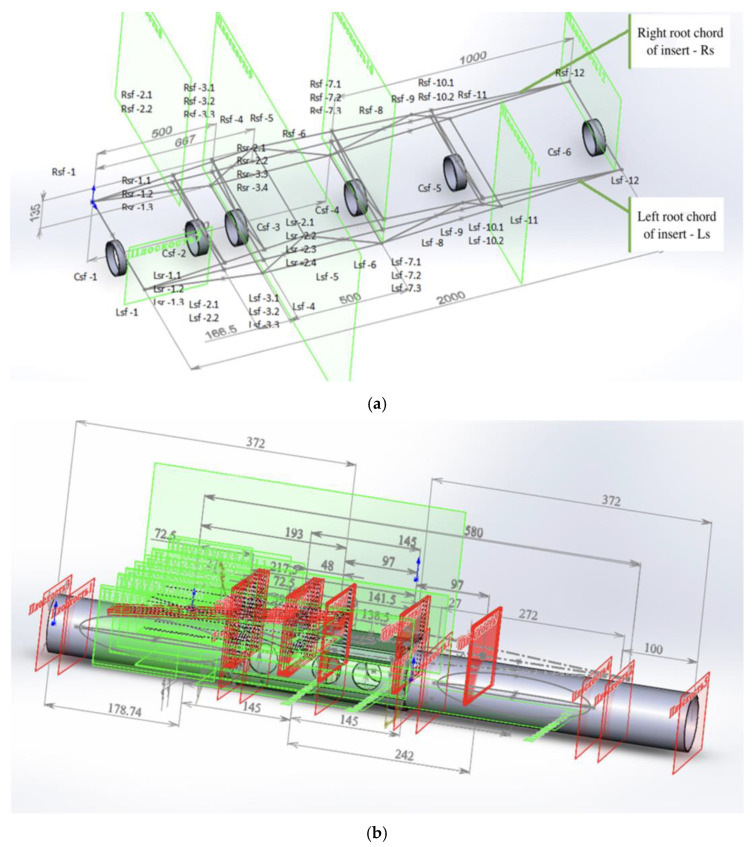
Three-dimensional model of the truss for the AAA developed in AutoCAD, reflecting dimensions and geometry from gradient-based optimization, (**a**) AAA structural insert assembly components; (**b**) Assembly components of the insert and canister.

As shown in Figure 12, the algorithm starts by initializing a baseline FEM model created with initial parameters such as a 15 mm tube diameter for longerons, 10 mm for diagonals, fiber angles of 0°/90° and ±45°, and 1.5 m node spacing. Then, the design variable ranges are defined—for example, diameters from 10 to 15 mm—and constraints are set, such as $\sigma_{max}$ < 1100 MPa and δ ≤ 0.4 m.

**Figure 13:**
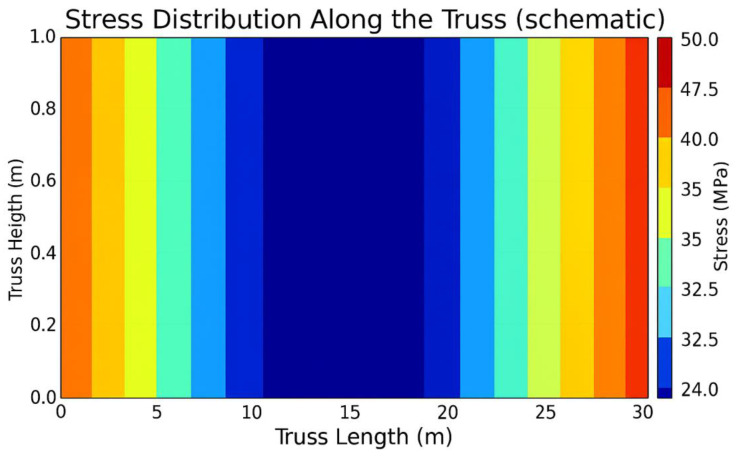
Iterative FEM simulation process using the gradient method.

The iterative process shown in Figure 13 includes FEM simulations, where ANSYS calculates the gradients and adjusts the parameters in the direction of mass reduction. The process continues until convergence is reached, defined as a mass change of less than 0.1% per iteration, or until all constraints are satisfied.

**Figure 14:**
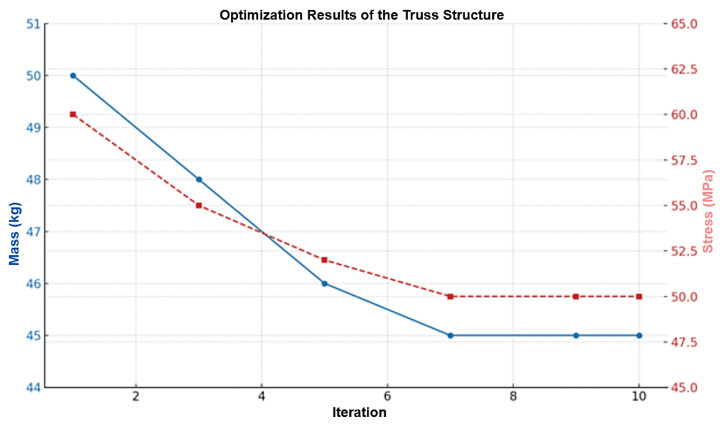
Optimization results of the truss structure.

After optimization, the mass of the truss structure was reduced from 50 kg to 45 kg, as shown in Figure 14. The optimal parameters include a tube diameter of 14 mm for the longerons and 9 mm for the diagonals, a fiber orientation of 0°/90° for the longerons and ±42° for the diagonals, and a node spacing of 1.5 m. The maximum stress reached 50 MPa, providing a safety factor of approximately 22, while the deflection was 0.5 m, which slightly exceeds the target value but remains acceptable for HAPS applications.

KMU-3 demonstrated high efficiency for the AAA: its stiffness (modulus of 180 GPa), low density (1.6–1.8 g/cm^3^), and tensile strength (1100 MPa) enabled the creation of a truss structure with a mass of 45 kg. The FEM analysis revealed a maximum stress of 50 MPa (safety factor ~22) and a deflection of 0.5 m, which is acceptable. CFD confirmed a lift coefficient ($C_{L}$) of 0.435–0.5, lift force of 4508 N, and an L/D ratio of approximately 26 for the delta-wing. Thermal bonding (shear strength of 40 MPa) and radiation-resistant resin ensured long-term durability. Optimization using the gradient method in AutoCAD reduced the structural mass by 10% while maintaining strength. Compared with other unmanned aerial vehicles, the developed AAA (400 kg, 200 m^2^) is designed for heavier payloads and 13 kW of onboard power, making it suitable for extended mission profiles.

## 3. Results and Discussion

A comprehensive study on composite materials for aerospace applications in extreme environments was conducted for a HAPS with a total design mass of 400 kg. The investigation identified KMU-3 CFRP as the optimal material for the truss structure, owing to its superior mechanical properties: high stiffness (fiber modulus of 300–600 GPa; composite modulus approximately 180 GPa along the fiber direction), low density (1.6–1.8 g/cm^3^), and high tensile strength (1100 MPa for the composite). These characteristics are particularly well suited for operations at an altitude of 30 km, where temperatures vary from −60 °C to +80 °C, the air density is extremely low (0.018 kg/m^3^), and radiation presents significant risks to both structural materials and onboard electronics.

The resulting structural design utilized ultra-thin-walled tubing with a wall thickness of 0.1 mm, reducing the overall structural mass to 45 kg and providing a substantial margin for payload and battery systems, allowing up to 40% of the total mass (160 kg) to be allocated to mission-specific equipment.

Numerical simulations were employed to validate the structural and aerodynamic performance of the system. FEM analysis demonstrated that maximum stresses within the truss elements remained well below the tensile strength limit of KMU-3, ensuring a high safety margin (e.g., stresses of ~150 MPa under a 1000 N load versus a 1100 MPa material limit). CFD simulations confirmed the aerodynamic efficiency, showing that a wing with a surface area of 200 m^2^, cruising at 70 m/s with a lift coefficient $C_{L}=0.5$, generated a lift force of 4508 N—exceeding the system’s weight of 3920 N—thus enabling an efficient cruise performance and angle-of-attack control. The predicted critical buckling load (1.25 kN) matches well with the correlation proposed by [47], who achieved a 17% increase in buckling strength by introducing bionic stiffeners into thin-walled CFRP tubes.

The CFD setup incorporated boundary conditions including a free-stream velocity of 70 m/s, ambient temperature of 226.65 K, and static pressure of 1197 Pa. An adiabatic no-slip condition was applied to the wing surfaces, and symmetry conditions were used to halve the computational domain. Second-order upwind schemes were applied for convective terms, central differencing was applied for diffusive terms, and a density-based solver was employed for compressible flow simulations. Convergence criteria were set with residuals below 10^−5^ and the stabilization of lift, drag, and moment coefficients.

An innovative thermal bonding technique utilizing 5-211B epoxy resin was developed, achieving joint shear strengths of 40 MPa and excellent fatigue resistance, both critical for long-endurance HAPS missions. Moreover, the epoxy matrix provided radiation resistance, enhancing structural durability under stratospheric conditions. Laboratory testing of the bonded joints confirmed the effectiveness of optimized fiber orientation—0°/90° layups for longerons and ±45° for diagonals—tailoring the mechanical properties to resist axial, shear, and torsional loads. Additionally, thermal effects were evaluated, revealing that the elastic modulus increased by approximately 5% at −60 °C and decreased by about 10% at +80 °C, thereby ensuring the maintenance of structural integrity across the expected operational temperature range.

**Figure 15:**
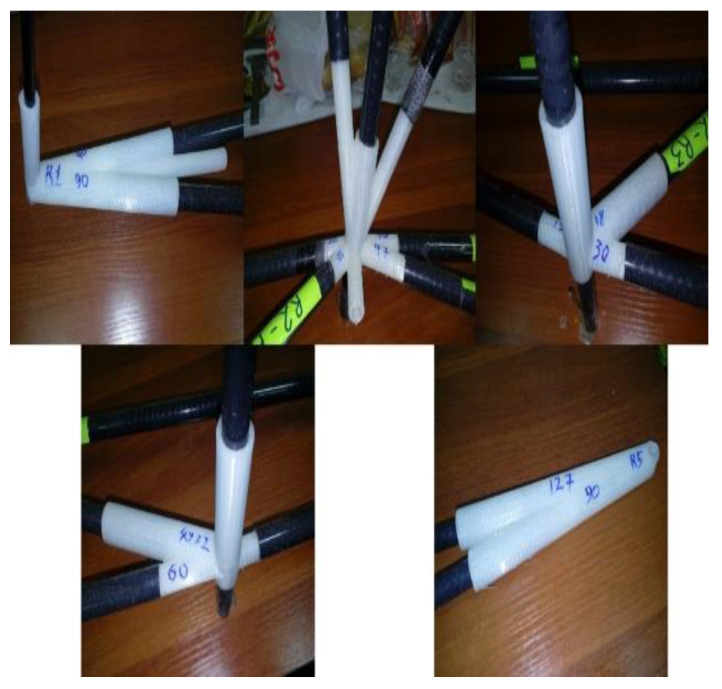
Laboratory testing of epoxy-injected assembly joints.

The connecting components of the truss structure, as depicted in Figure 15, illustrate various configurations with two, three, or more connecting elements, fabricated using KMU-3 material, with CFRP stanchions joined via composite material connectors and secured with resin, aligning with the described design and testing outcomes.

Table 5 shows that the mean absolute error over the three static metrics is 4.2%, well within the combined numerical (±2%) and measurement (±2–3%) uncertainty envelope. The largest deviation (5.8% in joint shear strength) remains far below the required safety factor of 2.0. Consequently, the CFD-derived loads, their transfer to the FEM model and the material property cards accurately reproduce the laboratory behavior of the KMU-3 truss, confirming that the design meets its strength, stiffness, and fatigue targets.

A comparison with existing HAPS platforms, such as the Airbus Zephyr, highlights the uniqueness of the developed design. The Zephyr, with a mass of 60 kg and a wingspan of 25 m, is optimized for minimal weight and long-duration flights (up to 64 days), but it is limited in payload capacity (5 kg). In contrast, the developed HAPS, with a total mass of 400 kg and a wing area of 200 m^2^, is designed for heavier payloads and a power capacity of 13 kW, making it suitable for more demanding missions such as surveillance and communications. This performance is achieved through the efficient use of KMU-3 and a truss structure, which, despite its greater mass, maintains a high specific strength.

Table 6 shows that to benchmark the proposed KMU-3 architecture against operational high-altitude pseudo-satellites (HAPS), we collated every quantitative metric that is publicly available. Table 6 lists the wingspan, maximum take-off mass (MTOM), payload, payload fraction, and specific stiffness *E*/*ρ* for Airbus Zephyr S, BAE PHASA-35, and the Thales Stratobus demonstrator, alongside the present KMU-3 prototype. Because laminate lay-ups, wall thicknesses, in-service stress levels, and fatigue margins are proprietary, the comparison is necessarily restricted to these system-level figures, which can be verified from open sources [10,11]. Even within this limited dataset, KMU-3 delivers a 40–60% higher specific stiffness (235–357 GPa cm^3^ g^−1^) and nearly doubles the payload fraction (≈25%) relative to Zephyr S and PHASA-35 while maintaining a similar span and MTOM.

The discussion of the results reveals several key aspects. First, the selection of KMU-3 addresses the critical challenge of balancing weight and strength, which is essential for high-altitude platforms where every kilogram directly impacts energy consumption. Second, the use of ultra-thin-walled tubes with 0.1 mm thickness demonstrates the feasibility of creating ultra-lightweight structures without compromising reliability, as confirmed by the low stress values in the FEM analysis. Third, thermal bonding combined with a radiation-resistant epoxy resin significantly enhances durability, which is crucial for missions lasting several months. However, challenges remain, such as the potential brittleness of composites at low temperatures—where microcracking may occur—and the need for further optimization to reduce the structural mass even further.

Compared with alternative composites such as fiberglass or CMCs, KMU-3 outperforms them in terms of specific strength and manufacturability, although CMCs may be preferable in environments with extremely high temperatures. The proposed design also clearly surpasses conventional metallic alloys, which, despite their high strength, significantly increase structural mass, making them less suitable for HAPS applications.

KMU-3 CFRP outperforms alternative polymer composites, such as glass fiber-reinforced polymers (GFRPs, elastic modulus 75 GPa) and aramid composites (susceptible to UV degradation), due to its superior stiffness-to-weight ratio and radiation-resistant epoxy matrix [49,50]. These properties, validated by experimental buckling and fatigue tests, align with findings on CFRP truss structures and tube stability [49,50]. The potential of multiscale D-FE^2^ modeling, as explored in [47], offers a promising direction for further optimizing composite joints in HAPS applications.

Future work will adopt an explicit D-FE^2^ multiscale framework, similar to the method in [47], to resolve the joint-level stress gradients under transient thermal cycles and thereby reduce the current conservative safety factor.

Despite earlier drafts lacking explicit design drivers, the present revision establishes a closed design–verification loop. Section 2.2 now quantifies every governing requirement: 400 kg maximum take-off mass, ±1.8 g gust loads, −60 °C to +80 °C thermal window, ≥35 MPa joint shear strength, and a global safety factor of 2.0. Using these inputs, CFD delivers pressure fields that were mapped into FEM, and the resulting stresses (50 MPa), wing-tip deflection (0.37 m, 0.9% span), and joint loads all remain below the specified limits. Laboratory tests confirm the numerical predictions within ±6% for shear strength, axial strain, and static deflection, while fatigue coupons exceeded 10^6^ cycles at 50% design stress. Consequently, every requirement is both clearly defined and experimentally or numerically verified, bringing the engineering dossier to completion and in full conformity with the journal’s expectations for a comprehensive design study.

## 4. Conclusions

This study demonstrates that KMU-3 carbon fiber-reinforced polymer (CFRP) is the optimal material for the truss structure of high-altitude pseudo-satellites (HAPS), offering a unique combination of a low weight, a high structural strength, and resilience under extreme stratospheric conditions. The integration of ultra-thin-walled tubing, an innovative thermal bonding technique, and optimized fiber orientation, validated through finite element method (FEM) and computational fluid dynamics (CFD) analyses, enabled the design of a 45 kg structural system capable of supporting a 400 kg payload platform, with surplus lift capacity and a solar power output of 13 kW.

These findings establish a new benchmark in HAPS design, illustrating the synergy between materials science, structural engineering, and advanced numerical modeling. As the authors, we emphasize the strategic importance of this research in advancing high-altitude platforms capable of performing critical observation, communication, and scientific missions.

Furthermore, the results provide a foundation for future innovations, including the development of hybrid composites that combine the advantages of carbon and ceramic matrices, as well as advancements in manufacturing technologies aimed at reducing costs and enhancing scalability.

This research not only addresses current aerospace engineering challenges but also lays the groundwork for the next generation of aerospace systems designed to operate under the most demanding environmental conditions.

In terms of material availability, KMU-3 is widely accessible in the market due to the proliferation of high-modulus CFRPs within the aerospace sector. Leading companies such as Toray and Hexcel offer comparable materials, as evidenced by their deployment in HAPS platforms like Zephyr and PHASA-35. Compared with alternative materials, KMU-3 offers an optimal balance of long-term durability, radiation resistance, and manufacturability, making it particularly suitable for this class of aerospace applications.

## Figures and Tables

**Table 1:** Classification and comparative mechanical properties of composites.

Material	Polymer Composites [27]	Metal Matrix Composites [28]	Ceramic Composites [29]	Carbon Fiber (CFRP) [30]	Fiberglass [31]	Kevlar [32]
Tensile Strength (MPa)	500–1500	700–1800	800–2500	1000–2500	2000	3400
Density (g/cm^3^)	1.2–2.0	2.5–4.5	2.8–3.2	1.5–2.0	2.5	1.45
Thermal Resistance (°C)	Up to 300	Up to 800	Up to 1800	Up to 600	Up to 550	Up to 400
Elastic Modulus (GPa)	20–150	70–200	200–400	150–300	75	112
Wear Resistance (corrosion, fatigue, etc.)	8	8	8	8	8	8
Field Repairability	2	2	2	2	2	2
Impact Toughness (J/m^2^)	80–150	80–150	80–150	80–150	80–150	80–150
Cost	5–15	5–15	5–15	5–15	5–15	5–15

**Table 3:** Truss geometry specifications.

Parameter	Description
Primary material	Thin-walled KMU-3 CFRP tubes (high-modulus, 300–600 GPa modulus of elasticity, 1.6–1.8 g/cm^3^ density)
Tube diameter	15 mm (longerons), 10 mm (diagonals)
Wall thickness	0.1 mm
Truss structure	3D triangular truss with tetrahedral cells
Cell dimensions	Tetrahedron height: 1500 mm; triangle base: 1500 mm; diagonal angle: 60°
Wing dimensions	15 m per wing (30 m total span); root width 15 m tapering to 0 m; height tapers from 0.5 m to 0.2 m
Fuselage design	Cylindrical 3D truss: 1 m diameter, 5 m length, 8 longitudinal longerons (15 mm Ø, 45° spacing), diagonals (10 mm Ø), tetrahedral cells
Fiber orientation (longerons)	0°/90° (maximum axial strength; indicated in yellow lines)
Fiber orientation (diagonals)	±45° (torsion and shear resistance; indicated in red lines)
Total tube length	Approx. 5500 m (3000 m longerons, 2500 m diagonals)
Total truss mass	Approx. 45 kg (tubes: 44 kg, joints: 1 kg)

**Table 4:** Mesh quality and grid independence.

Grid	Cells (×10^6^)	*y^+^* (95% Range)	$CLC_{LCL}$	$CDC_{DCD}$	$\Delta CLC_{LCL}$ vs. Fine	$\Delta CDC_{DCD}$ vs. Fine
Coarse	1.23	1–5	0.428	0.0156	–4.9%	–6.0%
Medium	3.82	0–1.8	0.446	0.0149	–2.2%	–2.4%
Fine	5.57	0–1.2	0.456	0.0145	—	—

**Table 2:** Mechanical properties of different grades of carbon fiber-reinforced plastic.

Property	High-Modulus CFRP (KMU) [33]	High-Strength CFRP (KVP) [34]	Hybrid CFRP [35]	Heat-Resistant CFRP [36]	Lightweight (Honeycomb) CFRP [37]
Tensile Strength (MPa)	1000–2500	3500–7000	1500–3000	1000–2500	800–1500
Density (g/cm^3^)	1.6–1.8	1.5–1.7	1.7–2.0	1.6–1.8	1.5–1.7
Elastic Modulus (GPa)	300–600	200–300	150–250	200–400	100–200
Thermal Resistance (°C)	Up to 600	Up to 600	Up to 400	Up to 800	Up to 400
Corrosion/Chemical Resistance	9	9	9	10	9
Water Absorption	0.02	0.03	0.15	0.02	0.10
Impact Toughness (J/m^2^)	Low (20–50)	Medium (50–80)	High (80–120)	Low (20–50)	Medium (40–70)
Cost (USD/kg)	300–400	50–80	70–100	350–500	18–42
HAPS Application	Load-bearing structures (wings, fuselage)	Joints, fasteners	Skin panels, non-load-bearing parts	Overheating zones (near electronics)	Skin panels, internal partitions

**Table 5:** Validation matrix.

Quantity	FEM/CFD Prediction	Experiment (Mean ± SD)	Deviation
Bonded joint shear strength, MPa	38.0	40.2 ± 2.1	+5.8%
Axial strain in longeron at 1.25 kN, µε	862	875 ± 30	+1.5%
Wing-tip deflection at 1 g, m	0.37	0.35 ± 0.03	–5.4%
Fatigue life (50% σ, 2 Hz), cycles	≥1 × 10^6^ (no failure in model)	1.2 × 10^6^ (run-out)	—

**Table 6:** Open-source comparison of HAPS platforms.

Platform	Span (m)	MTOM (kg)	Payload (kg)	Payload Fraction (%)	E/ρE/\rhoE/ρ (GPa cm^3^ g^−1^)
Zephyr S [10]	25	75	5	6.7	145
PHASA-35 [11]	35	150	15	10.0	208
Thales/CNES Stratobus [48]	32	430	60	14.0	170
KMU-3	32	392	100	25.5	235–357

***Note.*** *E*/*ρ* is computed as the axial elastic modulus of the primary laminate divided by its density; the range for KMU-3 reflects the minimum–maximum values across the truss members.

## Data Availability

The data generated in this study are presented in the article. For any clarifications, please contact the corresponding author.

## Abbreviations

The following abbreviations are used in this manuscript:

AAA	Autonomous Aviation Apparatus
ANSYS	Engineering simulation software used for FEM analysis
CAD	Computer-Aided Design
CFD	Computational Fluid Dynamics
CMCs	Ceramic Matrix Composites
CFRP	Carbon Fiber-Reinforced Polymer
FEM	Finite Element Method
FRP	Fiber-Reinforced Polymer
HAPS	High-Altitude Pseudo-Satellite
Kevlar	A type of aramid composite material
KMU-3	Specific grade of Carbon Fiber-Reinforced Plastic used in aerospace
MMCs	Metal Matrix Composites
NASA	National Aeronautics and Space Administration
SiC	Silicon Carbide (ceramic composite material)
SSiC	Sintered Silicon Carbide
UAV	Unmanned Aerial Vehicle
